# Caffeic acid: an antioxidant with novel antisickling properties

**DOI:** 10.1002/2211-5463.13295

**Published:** 2021-09-22

**Authors:** Tigist Kassa, James G. Whalin, Mark P. Richards, Abdu I. Alayash

**Affiliations:** ^1^ Laboratory of Biochemistry and Vascular Biology Center for Biologics Evaluation and Research Food and Drug Administration (FDA) Silver Spring MD USA; ^2^ Department of Animal and Dairy Sciences, Meat Science and Animal Biologics Discovery University of Wisconsin‐Madison WI USA

**Keywords:** antioxidants, caffeic acid, ferryl hemoglobin, sickle cell disease

## Abstract

It is well documented that caffeic acid (3,4‐dihydroxycinnamic acid) (CA) interacts with and inhibits the oxidative reactions of myoglobin (Mb) and hemoglobin (Hb), and this interaction underlies its antioxidative action in meat. Sickle cell hemoglobin (HbS) is known for its tendency to oxidize more readily than normal HbA in the presence of hydrogen peroxide (H_2_O_2_), which leads to a more persistent and highly oxidizing ferryl Hb (HbFe^4+^). We have investigated the effects of CA on HbS oxidation intermediates, specifically on the ferric/ferryl forms. At a low concentration of H_2_O_2_ (0.5‐fold over heme), we observed a fivefold reduction in the amount of HbFe^4+^ accumulated in a mixture of ferric and H_2_O_2_ solution. Higher levels of H_2_O_2_ (onefold and twofold over heme) led to a lesser threefold and twofold reduction in the content of HbFe^4+^, respectively, possibly due to the saturation of the binding sites on the Hb molecule. The most intriguing finding was that when 5‐molar excess CA over heme was used, and a considerable increase in the delay time of HbS polymerization to approximately 200 s was observed. This delay in polymerization of HbS is theoretically sufficient to avoid microcapillary blockage and prevent vasoconstrictions *in vivo*. Mass spectrometry analysis indicated that CA was more extensively covalently bonded to βCys^93^ than to βCys^112^ and αCys^104^. The dual antioxidant and antisickling properties of CA may be explored further to maximize its therapeutic potential in SCD.

## Graphical abstract

Caffeic acid (CA) is known for its antioxidant effects. The underlying mechanism of its action reported here includes reduction in the content of the highly reactive sickle cell ferryl Hb. CA also exhibited a novel antisickling effect by increasing the delay time before polymerization of HbS. Mass spectrometric analysis of the reaction reveals that CA covalently binds to the hot spot βCys93.

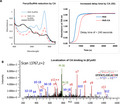

**Abbreviations**
2: 3‐DPG, diphosphoglycerate
5‐HMF: 5‐hydroxymethylfurfural
AA cells: normal RBCs
CA: caffeic acid (3,4‐dihydroxycinnamic acid)
FDA: Food and Drug Administration
H_2_O_2_
: hydrogen peroxide
HbFe^4+^
: ferryl Hb
HbS: sickle cell hemoglobin
Mb: myoglobin
NAD: nicotinamide adenine dinucleotide
NO: nitric oxide
ODC: oxygen dissociation curve
OECs: oxygen equilibrium curves
RBC: red blood cell
ROS: reactive oxygen species
SCD: sickle cell disease
SS cells: homozygous sickle RBCs
TD‐3: 4,4′‐di(1,2,3‐triazolyl) disulfide

Millions of people worldwide are inflicted with sickle cell disease (SCD) which has been recognized as a ‘molecular disease’ since the basis of the sickling process and the progression to organ damage have been well defined at the molecular level [1]. SCD is a genetic disorder in which a negatively charged amino acid (glutamic acid) is replaced by noncharged valine at position 6 in the β subunit of Hb. Under normoxic oxygen conditions where Hb is predominantly in the oxy form, this single amino acid substitution presents no conformational challenges to the Hb molecule or the red blood cell (RBC). However, under deoxygenating conditions in peripheral tissues, deoxyHb molecules tend to adhere together (as there is no longer repulsion due to the loss of a negative charge) through a sticky patch producing long fibers within RBCs. This results in rigid inflexible RBCs that are unable in some cases to navigate narrow capillaries leading to hemolysis and release of some Hb in circulation [2]. Polymerization of sickle cell hemoglobin (HbS) into long fibers is the fundamental pathology in SCD, and the development of small molecule drug agents that interfere with this process (directly or indirectly) has been an active area of research. These experimental approaches have been shown to some degree to minimize the polymerization of HbS and provided a useful therapeutic modalities [2].

Hydroxyurea (HU), the first approved product in the United States for use in SCD patients, increases HbF as well as it provides a secondary beneficial vasodilatory effect due to the formation of nitric oxide (NO) (byproduct of HU breakdown). l‐Glutamine is a precursor of nicotinamide adenine dinucleotide (NAD) and provides an antioxidant mechanism through the formation of reduced nicotinamide adenine dinucleotide (NADH) which has been approved by the Food and Drug Administration (FDA) in 2017. Voxelotor (previously known as GBT440), another FDA‐approved drug, modulates Hb function by increasing Hb‐oxygen affinity, thereby reducing Hb polymerization and sickling of RBCs [3]. Other reported classes of agents for treatment of SCD include aromatic aldehydes, thiol derivatives, isothiocyanates, and acyl salicylates and derivatives have been preclinically/clinically tested including the aromatic aldehyde, 5‐hydroxymethylfurfural (5‐HMF) which increases the affinity of HbS toward oxygen (for review, see [4]).

Besides the mechanical impediment created by HbS polymerization, RBCs are continually subjected to internal and external oxidative stresses. SCD RBCs are fueled by the presence of unusual levels of reactive oxygen species (ROS). These internal ROS mediate Hb’s own oxidative reactions which result in the formation of highly reactive intermediates such as the ferryl Hb (HbFe^4+^). Consequently, Hb undergoes extensive post‐translational modifications including irreversible oxidation of βcysteine 93 and the ubiquitination of βLys96 and βLys145 under oxidative stress conditions [5].

Oxidized βCys93, the target of ferryl radicals, has become a reliable biomarker of Hb, reflecting the deterioration of Hb within RBCs intended for transfusion or RBCs from patients with SCD. This led in the latter case to the designing of antisickling agents that can specifically target βCys93 providing therefore a dual antioxidant and antisickling therapeutic benefits in treating this disease. Molecules that specifically bind to or modify βCys93, such as 4,4′‐di(1,2,3‐triazolyl) disulfide (TD‐3) and hydroxyurea (HU)(by a process of S‐nitrosylation of βCys93), were shown to be more effective antisickling reagents when contrasted with molecules that target other sites on Hb molecule including 5‐hydroxymethyl‐2‐furfural (5‐HMF) and l‐glutamine [6].

The antioxidant properties of phenolic compounds such as caffeic acid (3,4‐dihydroxycinnamic acid) (CA) due in large part to its strong reduction potential and its ability to transfer electrons are well recognized. CA is synthesized by all plant species and is present in foods such as coffee, wine, tea, and popular medicines such as propolis. This phenolic acid and its derivatives have antioxidant, anti‐inflammatory, and anticarcinogenic proprieties [7]. It should be noted that dietary CA increased epithelial hyperplasia of the forestomach at ingestion levels of 678 to 2120 mg·kg^−1^ bw per day in mice and rats [8]. Thus, substantially lower pharmacological doses of CA should be evaluated in sickle cell animals’ models for safety and efficacy evaluation.

The novel interaction between CA and several animal Hbs and myoglobin (Mb) was reported in relation to its inhibitory action on lipid peroxidation as it impacts meant discoloration [9, 10, 11]. It is known that heme dissociation from metHb causes formation of free radicals that degrade protoporphyrin and cause lipid oxidation and that these reactions and crosslinking of heme to the protein were reported to be driven by small quantities of oxidant, HbFe^4+^ [12]. However, CA’s antioxidant action in preventing Hb‐mediated lipid peroxidation appears to be related to inhibition of heme release rather than direct inhibition of metHb formation [12].

Based on these early observations, we investigated the antisickling properties of CA, under oxidative stress conditions under which sickle cell Hb (HbS) is known to oxidize at faster rates and loses heme more readily than normal HbA. At fivefold CA concentration, there was a considerable decrease in the amount of HbFe^4+^ generated in the solution of ferric with H_2_O_2_ in addition to newly discovered antisickling properties (increasing the delay time before polymerization). These results are discussed in terms of potential application of CA in treatment of SCD with an advantage of being a powerful antioxidant.

## Materials and methods

### Materials

All chemicals and reagents were purchased from Sigma‐Aldrich (Saint Louis, MI, USA) or Fisher Scientific (Pittsburgh, PA, USA) unless otherwise specified. Blood samples used in this study were obtained with consent from homozygous sickle cell patients who are off hydroxyurea treatment during their routine clinic visits at the National Institutes of Health (NIH), and blood from healthy donors (homozygous for HbA) was obtained from the NIH Blood Center. Patients were enrolled after obtaining written informed consent in accordance with the Declaration of Helsinki on research protocols (03‐H‐0015 and 04‐H‐0161) that were approved by the Institutional Review Board of the National Heart, Lung, and Blood Institute (National Institutes of Health, Bethesda, MD, USA). Hb was purified as previously described using anion and cation chromatography [13]. Purified sickle Hb was obtained from Sigma‐Aldrich. AFSC control containing mixture of HbA, HbF, HbS, and HbC was purchased from Analytical Control Systems, Inc. Gases were purchased from Roberts Oxygen Company, Inc (Rockville, MD, USA). Rabbit polyclonal antibodies to SOD1 and catalase were purchased from Abcam (Cambridge, MA, USA). Hydrogen peroxide (H_2_O_2_) (30% w/w) was purchased from Sigma‐Aldrich. Fresh solutions of H_2_O_2_ were prepared for every experiment from a stock by making appropriate dilution in deionized water and kept on ice. The concentration of H_2_O_2_ was determined spectrophotometrically at 240 nm using a molar extinction coefficient of 43.6 M^−1^·cm^−1^ [14]. Buffer solutions were prepared by mixing solid monobasic and dibasic potassium phosphate dissolved in deionized water, and the pH was adjusted appropriately. DBBF was a gift from the US Army.

### Experimental procedures

#### Spectrophotometry and hemoglobin concentration measurements

Spectrophotometric measurements were carried out in a UV‐visible diode array spectrophotometer (Agilent HP 8453). The levels of ferrous, ferric, and ferryl Hbs were measured based on the absorbencies at λ = 541, 576, 630, and 700 nm using published extinction coefficients [13]. Hb concentrations were calculated on heme basis.

#### Hydrogen peroxide‐mediated oxidation and ferryl hemoglobin formation

Spectral changes due to H_2_O_2_‐mediated reaction of Hb (60 μm, heme) were monitored by photodiode array spectrophotometer using H_2_O_2_ (150 μm), in a total of 1 mL solutions at room temperature for 5 min of incubation times. Prior to treatment with H_2_O_2_, Hb was incubated with 5X (fivefold) caffeic acid at 0 °C for a period of 48 h. HbFe^4+^ formation (as a result of ferrous or ferric Hb oxidation) was followed by monitoring characteristic absorbance changes over time in the visible region. For the verification of the ferryl intermediate, 2 mm sodium sulfide (Na_2_S) was added to transform HbFe4+ to sulfhemoglobin (sulfHb). Formation of the sulfHb can be monitored by the appearance of an absorbance band at 620 nm and was estimated using published extinction coefficients [15].

#### Measurement of oxygen dissociation curves

Oxygen equilibrium curves (OECs) of RBCs and Hb solutions were obtained using the Hemox Analyzer^TM^ (TCS Scientific, New Hope, PA, USA). The oxygen dissociation curves (ODC) of suspensions of SS or AA cells were determined as previously described [16, 17]. To measure the ODC of homozygous sickle RBCs (SS cells) after treatment with caffeic acid, cells were washed, packed, and resuspended in plasma to a hematocrit of approximately 20%. The suspensions were incubated with 5 mmol·L^−1^ caffeic acid at 0 °C for 48 h. Approximately 120 μL of each suspension was added to 3 mL of Hemox buffer, pH 7.4, in a cuvette, and subjected to ODC analysis at 37 °C. A 120 μL of the 20% AA or SS RBCs suspension with 3 mL Hemox buffer was used as a control. Analysis was preformed using triplicate samples.

#### Polymerization kinetic measurements

Polymerization progress curves were carried out using a temperature‐jump technique to determine the effects of CA on the delay time [18]. HbS (1 mm) solution was treated with different concentrations of caffeic acid (CA) (2, 5, and 10 mm) prior to addition to the deoxygenated buffer solution as previously reported [16]. Temperature of the treated stock HbS solutions was then dropped to 0 °C by keeping the solution in an ice bucket. The reactions were kept at high concentration of phosphate buffer (1.8 M, pH 7.3) in a 1‐mL volume sealed cuvette with nitrogen gas bubbling for 60 min to deoxygenate the solution. A cold aliquoted solution of Hb (at 0 °C) was added to the cuvettes through a rubber septum with a Hamilton syringe to make final concentration of Hb in the cuvette as 120 μm. Then, the cuvette was placed in a preheated sample holder in the spectrophotometer (30 °C). The change in turbidity was measured with a temperature‐controlled photodiode array spectrophotometer at 700 nm. The optical density at 700 nm was plotted against time to give a typical sigmoidal curve characteristic of the progression in the delay time prior to the polymerization of HbS.

#### Electrospray ionization mass spectrometry (ESI‐MS)

ESI‐MS was performed at the Biotechnology Center (University of Wisconsin‐Madison). Analyses were carried out on a Orbitrap Elite mass spectrometer coupled to an IonMAX ionization source (Agilent, Palo Alto, CA, USA) with the sample injection as a 10 μL aliquot (0.5 mm Hb) at 30 μL·mL^−1^ flow rate from a 4.6 mm diameter autosyringe delivery system (Harvard Apparatus, Holliston, Massachusetts) in 50:50% methanol: water. Setting included capillary voltage 3500 V; drying gas 6.0 L·min^−1^; nebulizer 20 psig; gas temperature 325 °C; Oct DC1 39.5 V; fragmentor 180 V; Oct RF 250 V; Skimmer 60 V. Intact protein data analysis was carried out using Byos software from Protein Metrics (Cupertino, CA, USA) to produce isotopically resolved deconvoluted neutral masses, which were automatically matched against target protein sequences and a list of target masses for caffeic acid.

#### Tandem mass spectrometry with Orbitrap MS/MS

Hb peptides were prepared using trypsin/LysC with and without the use of iodoacetic acid (IAA) reagent. Dithiothreitol final concentration was 2 mm in the presence of IAA and 0.5 mm in its absence. Samples were desalted with a C18 OMIX tip. Samples were injected into a Pepmap C18, 3 μm, 100A, 75 μm ID, 1 5cm reversed phase column. Samples were analyzed on an Orbitrap Elite coupled to an EASY‐Spray ion source in data‐dependent MS/MS mode. Identification and quantitation of peptides was carried out using Byos software from Protein Metrics (Cupertino, CA, USA). After identification of peptides based upon their MS2 spectra, XICs were obtained based upon the MS1 m/z. % modification at residue positions was calculated based upon the normalization of the XIC AUCs of peptides, where multiple charge states were used. The same number of charge states were used for both modified and unmodified peptides.

## Results

### Caffeic acid lowers ferryl hemoglobin content

**Fig. 1:**
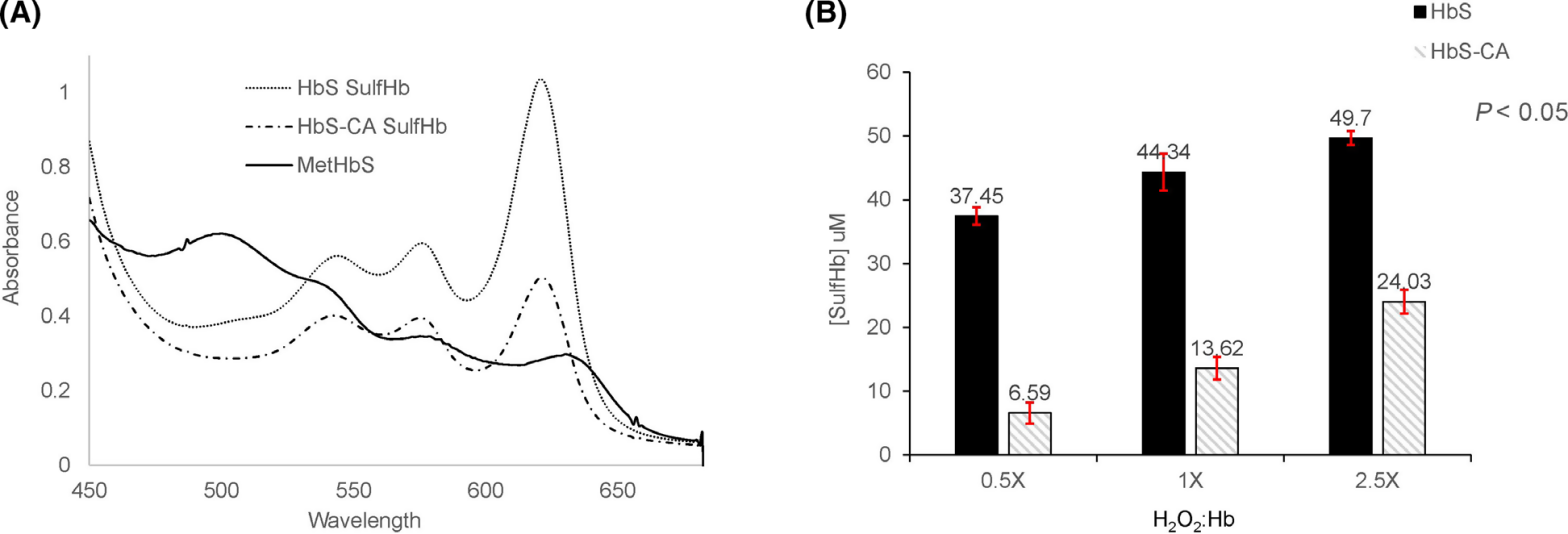
Spectral analysis of H_2_O_2_ oxidation of metHbS. Absorbance spectra were recorded before and after addition of 2 mm Na_2_S to 60 μm metHbS and 60 μm metHbS treated with 300 μm CA in the presence of 150 μm H_2_O_2_. Panel A shows the difference in the amount of sulfheme concentration in the presence and absence of CA. Panel B shows sulfheme concentration versus different H_2_O_2_ ratios to Hb. Error bars represent standard deviation.

The treatment of ferrous (Fe^2+^)/ferric (Fe^3+^) Hb with H_2_O_2_ transforms it to a higher oxidation state, ferryl (Fe^4+^) species and globin‐associated radical that can only be detected by EPR. The HbFe4+ spectrum is characterized by major peaks at 545 and 585 nm and a flattened region between 600 and 700 nm [19]. The ferric starting spectrum is characterized by two major peaks, 550 and 630 nm, respectively (Fig. 1A, solid line). The amount of HbFe4+ formed in the reaction was captured by the addition of sodium sulfide to convert the ferryl species to a spectrally more distinct and stable sulfHb, which has a characteristic maximum optical absorbance band at 620 nm. In Fig. 1A, overlaid sulfHb spectra of ferric HbS (60 μm) obtained with and without treatment by caffeic acid (300 µm) prior to the exposure to H_2_O_2_ (150 μm) are shown. The absorbance at 620 nm has clearly decreased with the treatment by CA, indicating that the amount of HbFe4+ formed has considerably reduced.

**Fig. 2:**
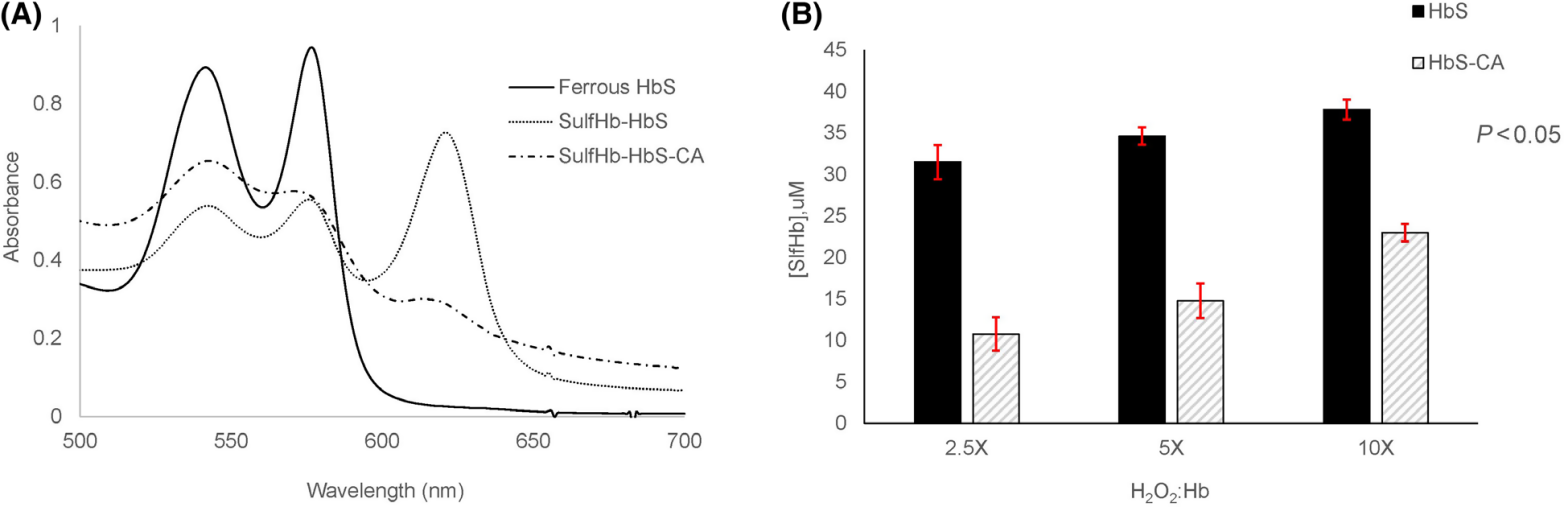
Spectral analysis of H_2_O_2_ oxidation of ferrous HbS. Absorbance spectra were recorded before and after addition of 2 mm Na_2_S to 60 μm ferrous HbS and 60 μm ferrous HbS treated with 300 μm CA in the presence of 150 μm H_2_O_2_. Panel A shows the difference in the amount of sulfheme concentration in the presence and absence of CA. Panel B shows sulfheme concentration versus different H_2_O_2_ ratios to Hb. Error bars represent standard deviation.

Figure 1B shows sulfHb levels in ferric (met) HbS solutions after treatment with CA in the presence of increasing concentrations of H_2_O_2_. For the Hb:H_2_O_2_ ratio (1 : 0.5), the sulfHb formed without treatment with CA resulted in 37.45 μm sulfHb whereas treatment with CA treatment resulted in 6.59 μm, almost fivefold reduction in the content of ferrylHb as measured by the sulfHb method. Similar trends of reduction of sulfHb amount were observed with higher ratio of Hb:H_2_O_2_ (1 : 1 and 1 : 2.5). SulfHb amount was reduced from 44.34 to 13.62 µm and from 49.7 to 24.03 µm for the 1 : 1 and 1 : 2.5 ratio of Hb:H_2_O_2_, respectively. We conducted similar experiments with ferrous HbS (Fig. 2A,B) and observed similar tends confirming the ability of CA to react with ferrous Hb and crosslinked Hb (DBBF), a diaspirin‐crosslinked Hb (developed as an oxygen‐carrying blood substitutes [20])(data not shown for DBBF).

### The impact of caffeic acid on ODCs

**Fig. 3:**
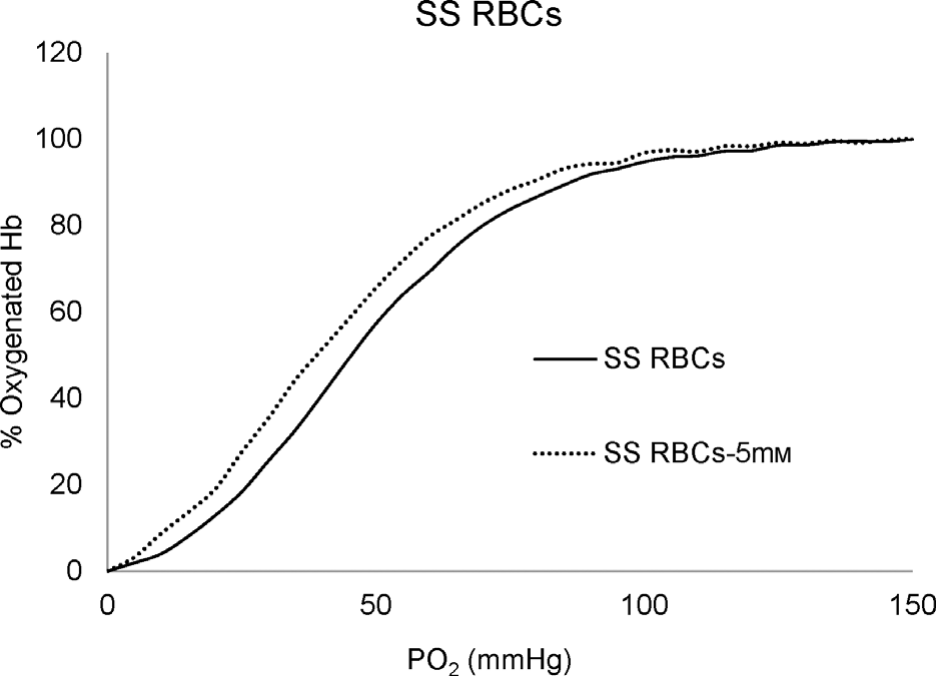
The effect of CA on the ODCs of SS intact cells. Suspensions of SS cell hematocrit (20%) were incubated in the presence of 5 mm CA at 0 °C for 48 h prior to determination of the ODC using Hemox Analyzer.

The ODCs representing suspensions of blood from SCD patient are shown in Fig. 3. With the treatment of CA, there was approximately 7 mmHg drop in the P_50_ (oxygen affinity when Hb is half saturated) values of SS cells with slightly reduced cooperativity in the ODCs (from *n* = 2.94 to 2.26). Importantly, the sigmoidal nature of the curve was largely maintained. CA is known to intercalate RBC membranes and modulate the properties of lipid membranes [21]. This led us to use considerably high concentration of CA (5 mm) for the RBCs assays compared with the 600 µm CA used for the free Hb (120 µm, heme) reactions. There was a small shift in P50s (2–3 mmHg) in HbA and HbS solutions (data not shown) compared with approximately (7 mmHg) in the RBC suspension. The P_50_ and *n* values for SS cells are summarized in Table 1.

**Table 1:** Effects of caffeic acid on the oxygen affinity of RBCs from a sickle cell patient. Suspensions of SS cell (20% hematocrit) were incubated with 5 mm CA at 0 °C for 48 h prior to determination of the ODC using the Hemox Analyzer. Control is a suspension of SS cells (20% hematocrit).

Caffeic acid (5 mm)	SS RBCs P50 (mmHg)	Hill coefficient (*n*)
SS RBCs (Control) (0)	46.27 ± 0.27	2.94 ± 0.06
SS RBCs + 5 Mm CA	39.56 ± 1.04	2.26 ± 0.11

### Caffeic acid increases the delay time before polymerization of sickle cell hemoglobin

**Fig. 4:**
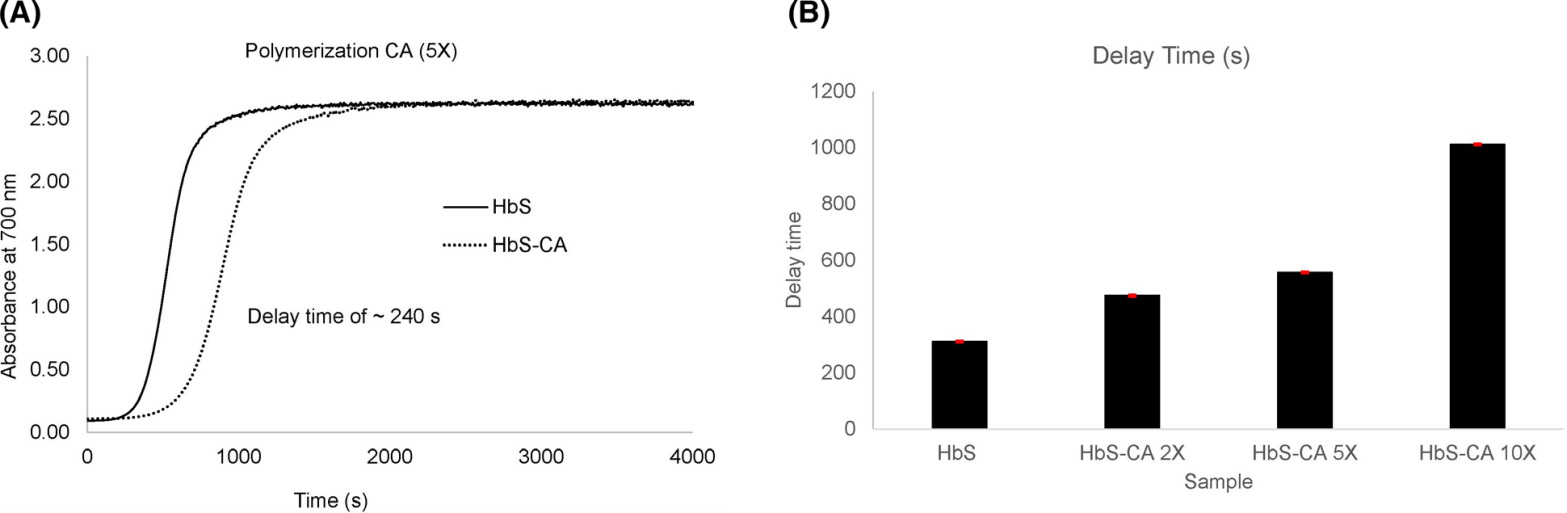
Effects of CA on the kinetics of deoxyHbS polymer formation. Solutions of deoxyHbS 120 μm were rapidly heated from 0 °C to 30 °C. Polymer formation was assessed by light scattering at 700 nm in 1.8 M phosphate buffer at pH 7.3. (A). Polymerization of deoxyHbS in the presence and absence of CA. (B) Bar graph showing the delay at various ratio of HbS:CA (1:0, 1:2,1:5, 1:10). Error bars represent standard deviation.

Deoxygenation of sickle Hb results in the polymerization of the Hb molecules into fibers that precipitate from the solution. Delay time is defined as the time between the beginning of deoxygenation and the appearance of the polymers [2]. The kinetic results reported (Fig. 4) were derived from temperature‐jump experiments measured during light scattering (at 700 nm) which reflects Hb polymerization. The absorbance at 700 nm at various time points was plotted as a function of time to generate typical sigmoidal curves shown in Fig. 4A as previously reported [22]. The figure shows a right shift in the curve indicating the increase in the delay time with the treatment of the HbS with CA. The delay time was increased by ˜ 247 s. The bar graph in Fig. 4B shows the delay time versus the HbS samples treated with various ratios of CA. The control HbS that was not treated with CA had a delay time of 310 s. The HbS solutions treated with 1 : 2, 1 : 5, and 1 : 10 ratio of CA had delay times of 474, 557, and 1012 s, respectively. Preferential binding to βCys93 stabilizes HbS in the high‐oxygen affinity R quaternary conformation, thereby shifting the T‐R quaternary conformational equilibrium toward R, which cannot enter the sickle cell fiber. These results show that aggregate formation or the polymerization of the deoxyHbS can be delayed with treatment of HbS with CA and confirms the antisickling property of CA.

### Mass spectrometric analysis confirms the specific binding of caffeic acid to alpha and beta cysteine residues

**Fig. 5:**
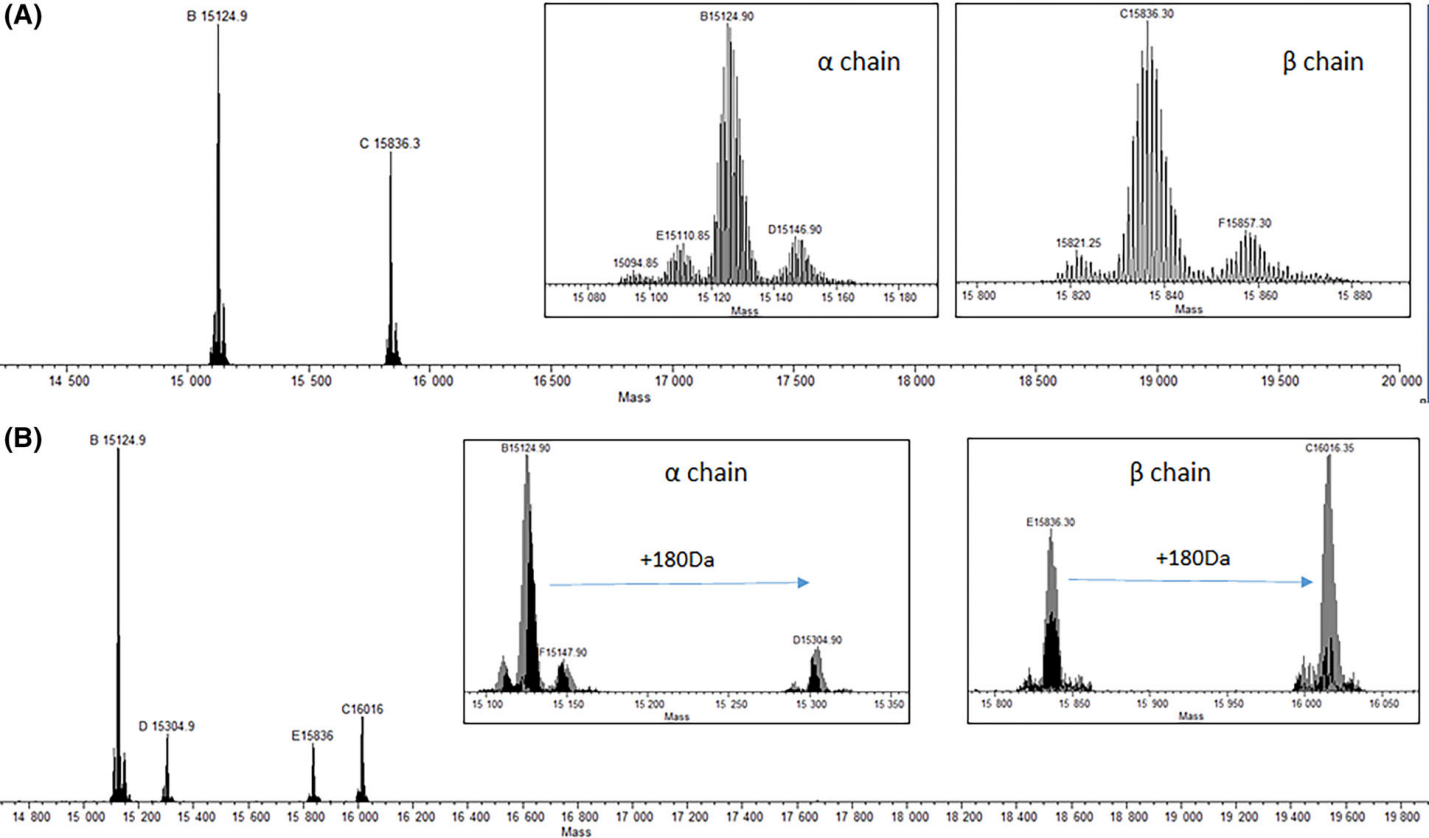
Deconvoluted ESI‐MS spectra of (A) HbS and (B) HbS + CA. The +180 m/z mass shift increase unique to HbS +CA in both the α‐chain and β‐chain indicates covalent bonding of CA to each HbS chain.

Solutions of HbS and HbS incubated with CA (1 : 10 ratio) for 48 h at 2 °C were submitted for ESI‐MS. The α‐chain and β‐chain of HbS were observed with masses of 15 125 Da and 15 836 Da, respectively, (Fig. 5A) as well as a + 180 mass increase on each chain after reaction with CA (Fig. 5B). This was indicative of one molecule of CA covalently binding to the α‐chain as well as one molecule of CA binding to β‐chain. After reaction with CA, there was less of the unmodified β‐chain relative to unmodified α‐chain (Fig. 5B) which suggested more extensive covalent binding of CA to the β‐chain. Quantitation of unmodified α‐chain was 88.6% compared with 47.6% unmodified β‐chain.

**Fig. 6:**
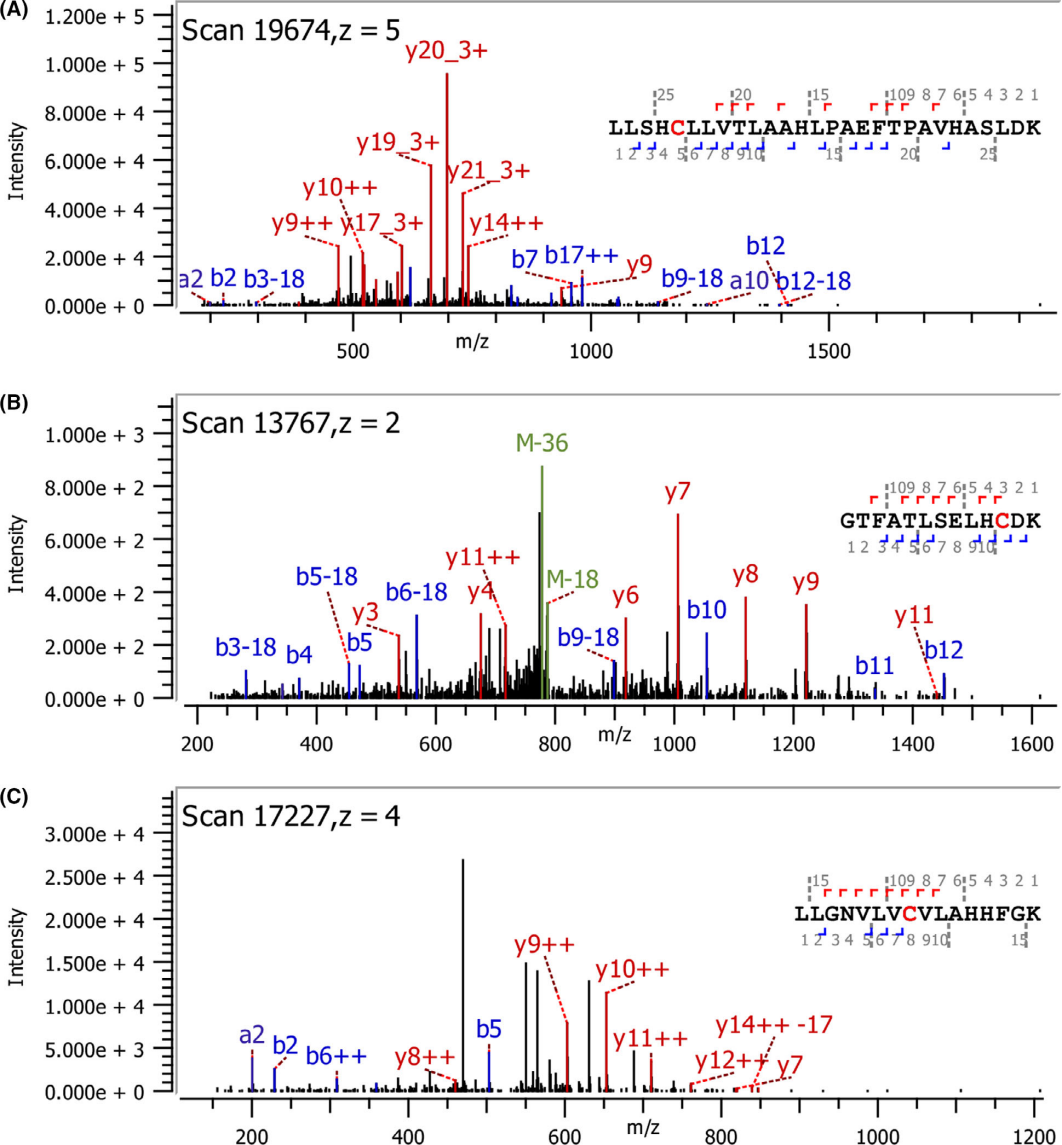
Localization of CA covalently bonded to Cys side chains of HbS using Orbitrap LC‐MS/MS. m/z plots of b‐ and y‐ions for (A) αCys^104^‐containing peptide, (B) βCys^93^‐containing peptide, and (C) βCys^112^‐containing peptide of HbS, each with a + 178 mass shift relative to the unmodified Cys‐containing peptide.

MS/MS analysis of digested HbS with and without CA exposure was done to examine site(s) of adduction. Exposure of CA to HbS resulted in a + 178 Dalton mass shift on a peptide containing αCys^104^, suggesting that CA covalently bound to αCys^104^, whereas no covalently bound CA was observed on β‐chain. It was suspected that DTT reagent may have dissociated bound CA from Cys residues of β‐chain. With this in mind, less digestion time, less DTT was used and alkylating agent (IAA) was excluded as a follow‐up to investigate bound CA on α‐chain and β‐chain. Based on MS2 ions, XIC retention times, isotope plots and delta mod scores, covalent bonding of CA to Cys of the βCys^112^‐containing peptides, to Cys of the βCys^93^‐containing peptides, and to Cys of the αCys^104^‐containing peptides was observed (Fig. 6). Further evaluation of the MS/MS data set indicated that CA was covalently bound to 99.1%, 59.8%, and 81.3% of βCys^93^, βCys^112^, and αCys^104^, respectively. The discrepancies in the degree of modification from intact protein analysis compared to that at the peptide level can be attributed to differences in ionization efficiency for each type of sample.

## Discussion

Among the most redox‐active phenolic compounds (e.g., CA, gallic acid, and tannic acid), known to vary in structure and number of hydroxyl groups, CA was found to be very effective in the inhibition of lipid oxidation in a washed muscle model system with added Mb and hemoglobin as oxidants [23]. The ability of CA to reduce metmyoglobin which involves thiols has been described [24]. CA inhibited heme loss from metHb potentially due to the binding of the phenolic acid to the Hb molecule [9] Mechanistically, it was suggested that increased access of protons to the heme crevice at the CD3‐H6P gap may result in protonation of the proximal histidine, weakening the heme‐globin linkage. A conformational shift due to CA binding could cause a shift of the amino acid at CD3 toward H6P and consequently decreases heme loss by improved blocking of solvent to the heme crevice [9]. Similar reduction kinetics of another hemoprotein, cytochrome c (covalently linked heme) by catechol(s), quinols, and related compounds such as hydrocaffeic acid have shown to act as a reducing agent through a rapid outer‐sphere mechanism involving the exposed heme edges in the proteins [25].

Because of CA’s redox action on hemeproteins in general and Hb and because of the proximity of its binding to the heme pocket, we explored its antioxidant effects on HbS, known for its venerability to oxidative side reactions, heme loss, and overall redox imbalance within SS RBCs. We also reasoned that CA, being a small molecule compound, may interfere with HbS fiber formation reducing subsequent hemolytic events in SS RBCs.

Although there are currently a few FDA‐approved drugs targeting mainly the downstream clinical sequel, very few small molecule compounds have been developed that target and inhibit intracellular Hb polymerization. There are multiple ways in which fiber formation can be inhibited and more recently molecular rationale for 5 distinct approaches to inhibiting polymerization was suggested. This includes, blocking intermolecular contacts in the sickle fiber, inducing synthesis of nonpolymerizing HbF, increasing oxygen affinity, reducing concentration of 2,3‐diphosphoglycerate (2,3‐DPG), and reducing intracellular Hb concentration [2].

There is increasing evidence for the role of oxidative stress in the pathophysiology of SCD [19]. ROS, derived internally as a result of Hb owns nonenzymatic autoxidative reactions such as superoxide ions, and from active enzymatic sources such as NADPH oxidase, were found in RBCs [26]. Additionally, SS RBCs unlike normal RBCs retained their mitochondria as they mature that can be an additional source for ROS [27]. This led to the suggestions that an improved understanding of oxidative stress will lead to targeted antioxidant therapies that should prevent or delay the development of organ complications in this patient population [28].

We have recently screened a number of small molecule drugs that exhibit dual antioxidant and antisickling properties and found that hydroxyurea currently approved for the treatment of SCD and a newly developed compound, TD‐3 exhibit antioxidant activity, by binding specifically to βcys93 preventing Hb oxidative instability [6].

Data reported here clearly demonstrate that CA has a strong antioxidant effect on HbS that surpassed the effects of other small drug molecules investigated so far. Moreover, CA demonstrated an antisickling properties by increasing Hb delay time for polymerization. The most noticeable effect was the ability of CA to considerably reduce HbFe4+ content formed during the reaction of peroxide and Hb. HbS oxidative side reactions, specifically the formation of a highly reactive ferryl, have been shown to drive cytosolic and membrane changes in SS RBCs taken from blood from sickle cell mouse model and from sickle cell patients [5]. At the molecular levels, HbFe4+ not only attacks other biological molecules but it targets its own β subunits, specifically, cystine 93 leading to its irreversible oxidation and heme loss [29, 30].

ESI‐MS of intact HbS indicated that one molecule of CA was bound to the α‐chain and one CA to the β‐chain, with more extensive binding to β‐chain. MS/MS analysis of the digested peptides at 2 mm DTT indicated that Cys^104^ of α‐chain was covalently bound with CA while CA bound to β‐chain was not observed. The Cys‐containing peptide of α‐chain showing bound CA was 28 residues in length. This may have created steric hindrance to DTT reductant in sample workup so that CA remained bound to αCys^104^, whereas DTT could reduce and thereby remove CA bound to Cys‐containing peptides of β‐chain (13 and 16 residues in length for βCys^93^‐ and βCys^112^‐containing peptide, respectively). Lowering the DTT concentration to 0.5 mm with decreased digestion time and removing IAA resulted in detection of CA covalently bonded to αCys^104^, βCys^93^, and βCys^112^. Oxidation of CA results in the formation of caffeoquinone that reacts with available thiols to form covalently bound CA [31]. Caffeoquinone formation takes place as oxidized equivalents of HbS are reduced by the added caffeic acid. Nucleophilic attack by the thiol toward the electrophilic quinone leads to covalently bound thiol at the 2' position of the benzene ring as was noted for methyl caffeate [32]. In addition, a greater degree of CA bonding to βCys^93^ and βCys^112^ was observed compared with αCys^104^ indicating selectivity toward β‐chain. The covalently bound CA to thiols of HbS may have contributed to the abilities of CA to decrease HbFe4+ formation (Fig. 2) and delay in HbS polymerization (Fig. 4).

As discussed earlier, one of the most common approaches to antisickling drug development is to identify a protein target and screen for a small molecule(s) that has a high affinity for a site on the surface of the HbS molecule that is involved in an intermolecular contact in the fiber [2]. CA showed no effects on oxygen binding affinity of free normal HbA and HbSS; however, incubation of CA with fresh RBCS from SS and normal subjects showed a modest left shift in Hb‐OECs. Generally, phenolic acids are known for their low lipophilicity and the lipid bilayer of cell membranes is predictably an entrance barrier hampering their direct intracellular activity [33]; however, it was reported that caffeic acid (20 mm) and quercetin (10 mm) were effective in preventing the cytotoxic effects of rat RBCs and more importantly facilitated the entry of CA to the inside of RBCs [34]. This may present a possible combination strategy to overcome poor permeation of RBCs by CA. Our data also showed that in addition to the antioxidant and antipolymerization effects, CA induced a moderate left shift in OECs toward less polymerizing high‐oxygen affinity R state in RBCs.

## Conflict of interest

The authors declare no conflict of interest.

## Author contributions

AA and MR designed the work. TK and JW performed the experiments and data analysis. AA and MR contributed to conception and design. AA, MR, TK, and JW wrote and edited the manuscript.

## Data Availability

The data will be available from the corresponding author upon reasonable request.
